# Understanding False Negative in Prenatal Testing

**DOI:** 10.3390/diagnostics11050888

**Published:** 2021-05-17

**Authors:** Mark I. Evans, Ming Chen, David W. Britt

**Affiliations:** 1 Fetal Medicine Foundation of America, Icahn School of Medicine at Mt. Sinai, New York, NY 10029, USA; evans@compregen.com (M.I.E.); dwbrit01@louisville.edu (D.W.B.); 2 Department of Obstetrics & Gynecology, Icahn School of Medicine at Mt. Sinai, New York, NY 10029, USA; 3 Department of Genomic Medicine, Changhua Christian Hospital, Changhua 50046, Taiwan; 4 Department of Obstetrics and Gynecology, National Taiwan University Hospital, College of Medicine, Taipei 10041, Taiwan; 5 Department of Biomedical Science, Dayeh University, Changhua 51591, Taiwan; 6 Department of Medical Sciences, National Tsing Hua University, Hsinchu 30013, Taiwan

**Keywords:** down syndrome screening, noninvasive prenatal testing, microarray, neural tube defect, mendelian screening panel

## Abstract

A false negative can happen in many kinds of medical tests, regardless of whether they are screening or diagnostic in nature. However, it inevitably poses serious concerns especially in a prenatal setting because its sequelae can mark the birth of an affected child beyond expectation. False negatives are not a new thing because of emerging new tests in the field of reproductive, especially prenatal, genetics but has occurred throughout the evolution of prenatal screening and diagnosis programs. In this paper we aim to discuss the basic differences between screening and diagnosis, the trade-offs and the choices, and also shed light on the crucial points clinicians need to know and be aware of so that a quality service can be provided in a coherent and sensible way to patients so that vital issues related to a false negative result can be appropriately comprehended by all parties.

## 1. Introduction

The COVID pandemic has publicized some of the difficulties in the interpretation of screening tests that clinicians and statisticians have been debating for decades. Anyone paying attention has had a crash course in understanding that a “test” is not always perfect, in fact, it does not always give the right answer, and that there are trade-offs in the accuracy and reliability of results [1]. Those trained in the discipline of screening will, of course, recognize the utilization of some of its basic principles to explain the current situation [2,3]. The majority of physicians and the general public seem confused by the apparent inability of the laboratories to give what was generally assumed to be simple yes/no answers to very important questions.

The same principles apply to all forms of screening. In prenatal genetics, common examples include aneuploidy, specifically, Down syndrome using multiple methodologies, Mendelian carrier screening, and pre-eclampsia [4,5,6]. On the gynecology side of obstetrics and gynecology, screening for malignancies of the cervix, uterus, ovary, and breast have developed rapidly (but are beyond the scope of this article) [7,8,9]. Before moving onto the nuances of emphasis that can be applied to how tests are designed and, equally-importantly, how cut-offs are arranged to favor either emphasis on sensitivity or emphasis on low false positive rates, we will first review some basics.

The first issue is to distinguish between screening and diagnostic tests. Simply put, screening tests only adjust odds, and diagnostic tests are meant to give a definitive answer (Table 1). Historically, there have been between seven and ten criteria that have been felt necessary to consider before deciding to screen for a condition [2,3,10]. We expand upon these and recategorize them to include issues of infrastructure, training, availability, acceptability, cost and political interference that before the COVID epidemic might have been taken for granted by those establishing guidelines for screening tests (Table 2).

Discussing these issues in any depth is beyond the scope of the present paper, but the context in which screening and testing takes place can no longer be taken for granted. In practice, not all tests being used, however, follow these guidelines, which can lead to disproportionate expectations (either positive or negative), expenditures, and complications from follow-up testing that are likely “unnecessary”. The goal of a screening program is to detect the maximum number of “affected” individuals for the least number screened false-positive. Specifically, a program is not judged by whether any particular patient’s problem can be identified [1,2,3]. Where to put the cut-off points in balancing these two is, by definition, arbitrary, but must be maintained to maximum efficiency.

Medicine, and particularly obstetrics, involves the repetitive use of both diagnostic and screening tests. Most patients and too many physicians do not understand the difference [1,2,3]. Diagnostic tests are meant to give a definitive answer, may have risks, may be expensive, and are only meant for patients at a risk high enough to warrant it. Conversely, screening tests are meant for “everyone”, and their aim is to divide a group with high-enough risk to warrant diagnostic testing from those who do not. They do not give definitive answers [1,2,3]. How well they do their job is defined by metrics of sensitivity, specificity, positive, and negative predictive value. These principles of evaluation were introduced into clinical practice in the 1970s by Galen and Gambino [1].

The performance characteristics establish the boundaries of a playing field and a scoring system within which tests compete for better ways to do things. Sensitivity and specificity as test properties are relatively well-known. Less often used are the criteria that summarize some of the critical trade-offs that clinicians face. One such trade-off is the relative number of true positive cases and false positive cases, which is reflected in the ratio of the two: True positives and false positives are defined as the Positive Likelihood Ratio (PLR). A second trade-off involves the relative number of false negatives and true negatives, reflected in the ratio of the two: False negatives and true negatives are defined as the Negative Likelihood Ratio (NLR). Competitive approaches should have PLRs that are high and NLRs that are far less than one and need to be “minimally fractional”.

Receiver operating characteristic curves are typically used to compare the efficacy of tests or combinations of tests by directly examining the trade-off between True Positives (Sensitivity) and False Positives (1—Specificity). In situations where positive cases are very rare, so that the data are unbalanced, Precision-Recall Curves are more useful because they are not biased by the overwhelmingly large number of negative cases [11] and instead they focus more attention on the proportion of positive cases.

Clinicians are in the position of having to take all of this information and present it in an understandable way to explain not only the prior risk of the patient, but also how that risk is changing over time using Bayesian principles [12]. In our experience, clinicians are more likely to see value in a screening test that changes a priori risk from 1/500 to 1/20 than one that changes it from 1/100,000 to 1/500 even though the likelihood ratio of the former is improved 10 times more in the former than in the latter. An explanation for this would be that in the former, a change in clinical behavior might occur whereas such change is less likely with the latter. We also previously reported that patients “suddenly” at risk (e.g., abnormal aneuploidy screening) had a higher “state of anxiety” over the situation than those with the same level of risk, but it was because of non-emergent risks such as advanced maternal age [13].

## 2. Choices

Given that, by definition, screening tests only adjust odds, there are options as to where to place the emphasis of the program. In our general approach to genetic counseling, we routinely tell patients that in most circumstances, for those in the “middle 98%”, it does not make a difference whether the patient has diagnostic testing for genetic abnormalities or not and everything will be fine. The question that we believe all patients need to understand is: “If you’re going to be wrong, which way would you rather be wrong? Would you rather take a small risk (e.g., a 2% risk of having a baby with a serious problem vs. a 0.1–0.12% risk of having a complication from a procedure in experienced hands) because you wanted to know that” [14,15,16]? We tell them that essentially the middle does not count.

The whole exercise then reduces to one question: “Tell me what you fear the most, then we can reduce that at the expense of the other options”. Unfortunately, encouraging patients to be active at the clinic level cannot be assumed to be representative across the board for at least three reasons. First, screening might be treated as part of routine medical care not “requiring” any patient decision-making processes [17], thus undermining the possibility that informed consent and shared decision-making would be part of the decision. Second, there may be a breakdown in shared decision-making owing to a various combination of class, racial-ethnic, or educational differences between genetic counselors and patients and the situation could create a collective fiction rather than shared decision-making [18]. Third, ideological differences may create at least a suspicion of genetic reasoning and analysis or outright hostility to it [19] or seek to create conditions in which genetic analysis assumes eugenic overtones [20,21].

**Figure 1:**
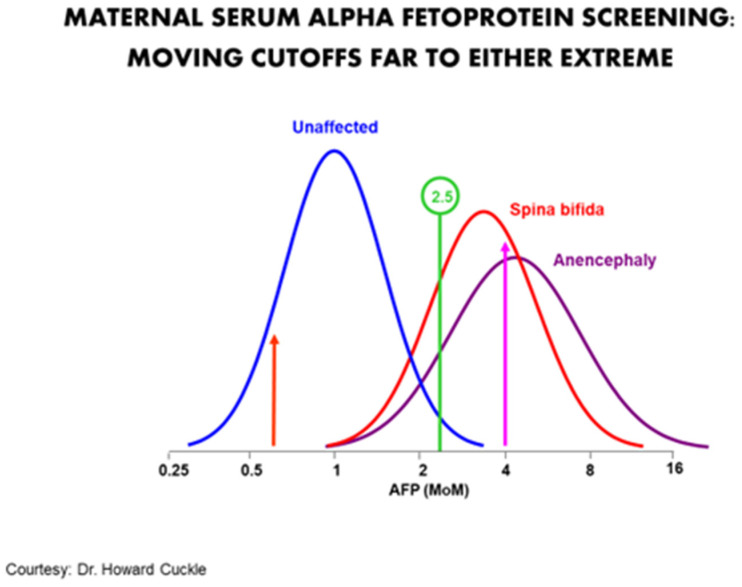
Typical cut-off of 2.5 MOM identifies about 90% for a 5% false positive rate. Moving the cut-off to 4 MOM would significantly increase the positive predictive value in an abnormal, but would also result in many false negatives. Moving it far to the left (about 0.6 MOM) would increase the sensitivity by almost 100% but at the price of having a screen positive rate of nearly 70%.

The purpose of a screening program is not to maximize the correct diagnosis of any given patient. Rather it is to maximize the collective detection of problems for the least number of false positives. It can also be thought of as mimicking the ethical principle of proportionality, i.e., obtaining the most benefit for the least harm. A particularly good example was the use of maternal serum alpha-fetoprotein (MSAFP) for neural tube defects (NTDs). In the 1970s to the mid-1980s, the standard cut-off of 2.5 multiples of the median identified about 90% of NTDs for about a 5% false positive rate. Moving the cut-off to the right would increase the positive predictive value at the expense of sensitivity. Moving it to the left would increase the sensitivity but also create many more false positives (Figure 1). There is no absolute clarity as to where on the continuum the optimal cut-off point should be.

**Figure 2:**
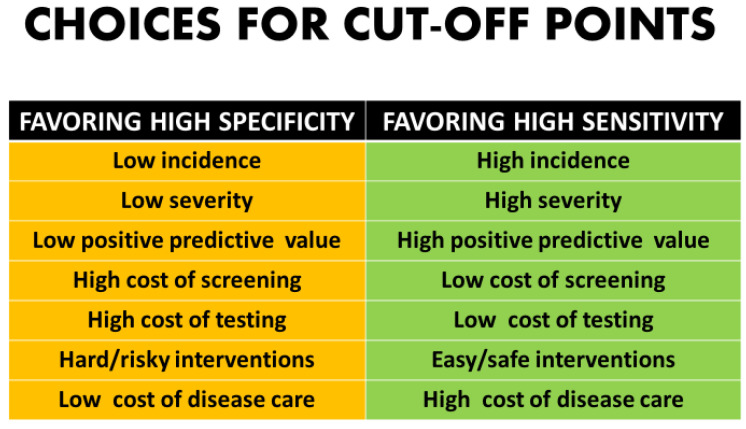
Choices for cut-off points.

Whether to maximize sensitivity or keep the screen positive number to a minimum involves not only mathematical analysis but clinical and ethical value judgements [2,3] (Figure 2). There are many components in the decision-making process for patients and couples as to whether they desire diagnostic testing.

Given the ethical, religious and politically charged nature of prenatal diagnosis and its relationship to the abortion issue, it is not surprising that there are vast differences in the utilization of such technologies by regions of the world. Within large countries such as the United States, variations occur as a result of political affiliation, religious beliefs, social class and education, as well as multiple other personal and traditional “sociological” variables.

Policy makers have to make decisions about where to put cut-off points. Setting policies may be decided by the program directors, government officials, or insurance companies. They may be uniform throughout a country or vary enormously even in the same environment. Alpha-fetoprotein (AFP) diagnosis from amniotic fluid was developed in 1972. At that time, the recurrence risk in the United Kingdom for a couple with one prior NTD was about 3–5% and, with 2 prior NTDs it was nearly 10%. Such patients were directly referred for amniocentesis even though its risks were thought to be nearly 2%, and because ultrasound was non-existent [22]. While recurrence risks were much higher than the primary incidence, 95% of all NTDs occurred in couples with no prior or family history [23,24]. The overall primary incidence of NTDs in Scotland was about 1/400. Given the high risk of procedures at the time, a screening approach was the only realistic public health measure to detect a highly significant or highly prevalent disorder. MSAFP was first introduced in 1973 as a screening test to determine who, among the supposedly low risk group, was at high risk [25].

The MSAFP cut-off was determined based upon conversations between the laboratory and the obstetrics department in Edinburgh to model what cut-off point was required to produce, by identifying and ranking the highest risk patients, the maximum number of “at risk” patients that could be accommodated by the clinical service at the hospital. Having the resources available is a relevant and rational contextual element is purely rational but assuming that the political process will operate in a way that is driven only by risk can no longer be taken for granted. Extending this argument, one can no longer assume that tests will not be reserved for those of higher status, or, on the other side, that tests will not be routine for those with lower status. At the most general level, neither micro-contexts nor macro-contexts [26] may be assumed to be immune from political influence.

Implementation has also been problematic. In 1988 we reported enormous variation in MSAFP screening in the Detroit area such that the same specimen could be interpreted by laboratories across the full spectrum from being very high risk to very low risk. Likewise, some laboratories stated the need for certain parameters to be known (such as maternal weight and ethnicity), but then did not insist that they be provided, or even include them in their analysis [27]. It took decades to coalesce on quality control measures to ensure better standardization of the accuracy of measurements and the relationship between the raw measurement and its clinical interpretation. In the 1970s, it was common belief that laboratories in different cities needed to have their own reference ranges for MSAFP because the populations were “different” [28]. Eventually, it was determined that the discordance was, to an exceptionally large extent, a function of different laboratory methodologies. When standardized across the country the differences between cities completely disappeared. Racial and ethnic differences still remained, but they alsodid not vary across cities.

In the mid-1980s, as MSAFP screening moved from just NTDs (high MSAFP) to adding Down syndrome and Trisomy 18 (low MSAFP), assay changes were required to increase the accuracy of low-level measurements [29]. One effect was a distribution shift such that the percentage of cases with high MSAFPs fell from about 5% to 2.5%. Given that the majority of NTD cases had MSAFP values over 4.0 multiple of the median (MoM), the change resulted in very few additional false negatives and simultaneously improved the positive predictive value of those cases at risk [30]. Increasing the quality of ultrasound technology and clinical expertise also mitigated the risks of missed MSAFP screens [31].

Likewise, as MSAFP expanded to double (MSAFP + total human chorionic gonadotropin (hCG), then with some centers substituting free β hCG for total), triple (adding estriol), and eventually quad(adding inhibin), there were many arguments over the best combination with most arguing about estriol [32]. Arguments between the “double” vs. the “triple” supporters reached almost religious fervor. (We belonged to the “double” religion). Ultimately, analysis showed that whereas variables such MSAFP and the hCGs had stable coefficients of variation (COV) at around 5–7%, the estriol assay was much less accurate [32]. Some laboratories reported a similar 5–7% COV and demonstrated that estriol added value. However, for many laboratories, the COV was as much as 30%, rendering estriol useless or worse [32].

With the introduction of nuchal translucency (NT) measurements, the COV problem became considerably worse. In order to control for highly variable quality and accuracy, the Fetal Medicine Foundation in London developed training and screening reviews and certifications and showed increasing quality world-wide [33]. Later the Nuchal Translucency Quality Review (NTQR) program of the Society for Maternal Fetal Medicine in America introduced training programs in the United States. We reported that the Fetal Medicine Foundation program seemed to generate higher quality control than NTQR which lead to higher sensitivities and specificities [34,35]. We developed an approach to “handicap” less proficient providers by modulating how much those measurements counted in the algorithm [36]. It worked but never made it into the mainstream as the concurrent development of cell free fetal DNA came into practice whose protocols almost eliminated the use of NT measurements for Down syndrome screening in the practice landscape of prenatal diagnosis and obstetrics.

The two situations above (COV of estriol and NT quality) illustrate the conundrum of trying to improve the statistical performance of screening by insisting upon quality controls. It can be hard in the laboratory; it is much harder for more subjective variables, e.g., ultrasound NT measurements, with multiple clinicians involved [33,34,35,36]. Each provider has their own strengths and weaknesses, and there is well known variability among providers [37]. These types of situations emphasize further issues of gradient between absolute conformity of all care and widespread individuality that can elevate some providers but reveal weaknesses at the same time. The former elevates weaker providers but inhibits excellence at the top end.

Once Down syndrome became the primary focus of screening, multiple generations of improvements have occurred. The shifts from MSAFP to double, triple, and quadruple screening in the second trimester and NT, free β hCG, pregnancy associated plasma protein A (PAPP-A), combined screening, cell free fetal DNA (cffDNA) in various forms in the first trimester have all had the underpinnings of simultaneously improving both sensitivity and specificity [38,39,40]. Ironically, however, the improved screening for Down syndrome has come at the cost of increased failures to detect other problems that could have been found by using high quality ultrasounds and diagnostic procedures such as chorionic villus sampling with improved laboratory tests such as microarrays that can find, particularly in younger patients, 10 times the abnormalities that can be done by cffDNA [14,15,16].

Changes in the perceived efficacy of tests can have wide ranging effects upon the practice of medicine and society. On the positive side, improvements in Mendelian Screening have allowed the identification of at-risk couples to occur BEFORE they have experienced the impact of an affected child. Likewise, most fetal cardiac anomalies can now be detected prenatally allowing births to be moved to centers capable of advanced care rather than having to have panic neonatal transfers or no transfers at all [41,42]. Unfortunately, the rate of incorporation of new approaches is highly variable among industries. Medicine has historically been on the slower end of the spectrum and obstetrics on the slower end of medicine [43]. NT screening is a good example. It was adopted much faster in the UK and Europe than in the USA [37,43]. Likewise, there is a pattern in which there is first the development of new technologies followed by diffusion out to the community. As new methods expand, the utilization increases, but complications can increase rapidly until there is time for community understanding and education [44].

The sociological and societal implications of such understanding have dramatic implications for the practice of medicine and the health of the population. Due to the increased “screening” for Down syndrome there has been diminished utilization of procedures that we have described as an epidemic of MISSED abnormalities because of cffDNA. The public health effect has meant more unintended babies with serious disorders that, alongside other issues, have expensive care requirements [14,15,16].

Even getting past technical, financial, implementation issues, and quality controls, there are still philosophical issues that drive the determination of a cut-off point. The more serious the disorder, the more likely that any typical patient’s “tolerance” for missing a case would be very low [45,46]. This would create an impetus to put the cut-off point “to the left” on the distribution curve which would maximize sensitivity at the expense of increased false positives. Likewise, with disorders with a very low incidence or those in which morbidity and mortality might be moderate, it would be reasonable to move the cut-off to the right to minimize the number of patients undergoing follow-up diagnostic procedures [1,2,3] (Figure 2).

We have previously modeled the detection of genetic disorders comparing those identifiable by cffDNA versus diagnostic testing using chorionic villus sampling or amniocentesis with enhanced genetic testing using array comparative genomic hybridization (aCGH or microarrays) [14,15,16]. We reported that the yield of detected abnormalities was about 10 times greater using the diagnostic testing vs. screening. While this is to some degree an apples vs. oranges comparison (screening vs. diagnostic), it does highlight the trade-off of simplicity and lower cost versus comprehensiveness and greater expense for the detection and management of rare but significant outcomes. More direct examples within the screening world would be karyotype vs. microarray, small ethnically derived Mendelian screening panels vs. larger pan-ethnic panels, and Pap smears and mammograms vs. BRCA and Lynch syndrome molecular panels [7,8,9].

Some prenatal diagnosis centers are known for their procedure acumen. Patients who want “definitive” answers tend to go to such centers, often directed by referring physicians sensitive to their patient’s desires [14]. Conversely, other centers are known for a limited use of diagnostic procedures. To a degree, everyone “votes with their feet” as to where they go. A continuing challenge is assuring that patients have sufficient access and understanding to make informed “foot” (certainly autonomous) choices.

For some parameters, such as advanced maternal age, there are at least de facto standards as to what the borderline is. In the United States, advanced maternal age was 40 in the early 1970s, then fell to 38, and has been 35 for over three decades [3]. However, screening has used the cut-off as a starting point. For methods such as MSAFP, multiple markers and ultrasounds in the second trimester, and combined screening and nuchal translucencies in the first trimester, statistically use a “likelihood ratio” to multiply (×) the screening result with the a priori risk of achieving a risk which is then compared to the a priori risk [34,36]. The situation with cffDNA is more complicated as some laboratories use an absolute, (i.e., we see it, or we do not), and others base their risk prediction estimate on the background risk as defined by maternal age. Some vary their risk certainty by the fetal fraction of the specimens, and others do not [47]. These variations in style occur as physicians and clinics adapt to the needs, preferences and cultures of the populations they serve. The systemic challenge is to continually update both training and the diffusion of more effective technologies.

cffDNA testing was originally publicized by its developers to be a diagnostic test. The acronym NIPD for noninvasive prenatal diagnosis was commonly used. However, it soon became clear that “NIPD” was only a screening test based upon calculation no matter which methodology was chosen [48].

Fetal cell testing was stopped when cffDNA was developed. Recently, some researchers have tried to revive the cell-based non-invasive prenatal testing (cbNIPT). There are theoretical advantages of cells vs. cffDNA. Cell-based methods would give a more direct confirmation of fetal vs. maternal DNA and could have faster whole-genome applications. If so, the cell-based testing could be the solution to creating a diagnostic rather than a screening test and thus be capable of reducing false negatives inherent in cffDNA prenatal testing.

Some early cbNIPT studies (1970s and 1980s) were based upon capturing trophoblasts, which intuitively cannot solve the limitation of fetoplacental mosaicism [49,50]. In the 1990s and early 2000s, most approaches, including the National Institute of Child Health’s “NIFTY study” used nucleated erythrocytes, but the technologies were not robust enough [51]. Newer methods using fetal nucleated red blood cells (fnRBCs) might be able to solve the limitation of fetoplacental mosaicism which is inherent in both trophoblast-based cbNIPT and cffDNA testing. However, the possible revival of cbNIPT, even with the utility of fnRBCs instead of trophoblasts as the diagnostic target, will be built upon the prerequisite to solve the long-existing limitations of the technologies themselves: questionable reproducibility, consistency, scalability, and even reliability should be improved first. Automation by incorporating artificial intelligence (AI) to reduce manual work may be a way to help, and the progress in the highly similar field of circulating tumor cells (CTCs) can be referenced [52]. Much more work, including large-scale validation studies, is needed to verify its feasibility [53,54]. Supposing such efforts by capturing fnRBCs materialize, the problem of true fetal mosaicism is still beyond the scope of such technology.

We raise this issue to highlight the next generation “tug of war” between screening versus diagnostic approaches that we have previously addressed between cffDNA and microarrays [14,15,16]. Well-informed genetic counseling will be critical before utilizing any emerging new genetic testing, either screening or diagnostic. Considerable evidence suggests that the vast majority of obstetricians world-wide are not well-versed in the new technologies [14,15,16,47,55]. The more reliable a test is, the less dependent health care delivery will be on resources that do not exist for a very high-level clinical interpretation of data.

## 3. Conclusions

A diagnostic test is meant to give a definitive answer. Screening tests only alter odds. There are many assumptions and decisions that have to be made arbitrarily by the program directors that profoundly influence the outputs and can be swayed towards one extreme (maximizing sensitivity) or the other (minimizing screen positives). Even minor shifts in screening program philosophy and practice can have enormous public health implications.

Most programs do not publicly report these underlying decisions, or how their operations conform or vary from other laboratories in their area. Most programs likewise do not declare their endpoints in situations in which outcomes are not clear-cut and how such end point uncertainty colors their predictive values. There is no likelihood that there will be any uniformly agreed-upon strategy for such decisions, but clinicians should at least be aware of these issues and how they affect the performance of all screening tests. Understanding the scientific and sociological contexts of false negative results, in addition to false positive results, will be very important when clinicians are interpreting the results of prenatal screening and diagnostic tests and thereby can offer counseling to their patients with acceptable and decent quality. The misconception of confusing a screening test with a diagnostic one will bring negative impacts to the overall obstetric practice and the populations they serve, as we have seen in the recent history when cffDNA-based NIPT was introduced since 2011 [14,15,16].

## Figures and Tables

**Table 1:** Diagnostic vs. screening tests.

Screening Tests	Diagnostic Tests
Meant for everyone	Only done on “at risk” patients
Only adjust odds and do not give a definitive answer	Meant to give a definitive answer
Tests typically have little risk	Tests may have some procedural risks
Typically less expensive	Typically more expensive

**Table 2:** Criteria for effective screening and testing programs.

Disease	Screening	Testing	Intervention
High enough incidence	Good performance metrics	Good performance metrics	Beneficial intervention possible
	Availability and affordability of screening	Availability and affordability of testing	Availability and affordability of intervention(s) at different levels
	Acceptability of screening	Acceptability of testing	Acceptability of intervention(s) at different levels
Impairing or fatal	Capacity for follow-up and feedback	Capacity for follow-up and feedback	Benefits outweigh risks
Adequate political support and coordination for public health	Adequate political support and coordination for screening	Adequate political support and coordination for testing	Adequate political support and coordination for interventions
